# The incision strategy for minimizing sural nerve injury in medial displacement calcaneal osteotomy: a cadaveric study

**DOI:** 10.1186/s13018-019-1411-7

**Published:** 2019-11-12

**Authors:** Jeong-Hyun Park, Kwang-Rak Park, Digud Kim, Hyung-Wook Kwon, Mijeong Lee, Yu-Jin Choi, Yong-Been Kim, Suyeon Park, Jinseo Yang, Jaeho Cho

**Affiliations:** 1 Department of Anatomy & Cell Biology, Graduate School of Medicine, Kangwon National University, Kangwon, Republic of Korea; 2 Department of Orthopaedic Surgery, Chuncheon Sacred Heart Hospital, Hallym University, 77, Sakju-ro, Chuncheon-si, Gangwon-do 200-704 Republic of Korea; 3 Department of biostatistics, College of Medicine, Soonchunhyang University Hospital, Seoul, Republic of Korea; 4 Department of Neurosurgery, Chuncheon Sacred Heart Hospital, Hallym University, Chuncheon, Republic of Korea

**Keywords:** Cadaver study, Calcaneus, Osteotomy, Incision, Sural nerve

## Abstract

**Background:**

The skin incision for medial displacement calcaneal osteotomy (MDCO) often damages the sural nerve. We aimed to identify the practical reference area in which the surgeon can incise the skin to minimize the injury of the sural nerve during MDCO.

**Methods:**

The foot and ankles of 20 cadavers were dissected. The landmarks were the following four anatomical references: point A, the tip of the lateral malleolus; point B, the inferior margin of the calcaneus on the vertical line through point A; point C, the posteroinferior apex of the calcaneus; and point D, the lateral border of the Achilles tendon on the horizontal line through point A. The distances from the sural nerve to points A and B in the vertical direction (lines D1 and D2, respectively), to points A and C in the diagonal direction (lines D3 and D4, respectively), and to points A and D in the horizontal direction (lines D5 and D6, respectively) were measured.

**Results:**

The median ratios of D1 to D1+D2, D3 to D3+D4, and D5 to D5+D6 were 0.34 (range 0.25 to 0.45), 0.23 (range 0.16 to 0.33), and 0.38 (range 0.26 to 0.50), respectively.

**Conclusions:**

The distance ratios according to easily identifiable references would be a more practical incision strategy for surgeons to minimize sural nerve injury in both open and minimally invasive/percutaneous MDCO.

## Introduction

Medial displacement calcaneal osteotomy (MDCO) corrects hindfoot valgus by increasing the medial pull of the Achilles tendon by moving the posterior part of the calcaneus medially [15]. Historically, this procedure was the most common method to treat adult acquired flatfoot deformity (AAFD) with stage II tibialis posterior tendon insufficiency, in combination with other surgical procedures [8]. The osteotomy is usually performed in a direction perpendicular to the calcaneus axis or 45° to the plantar aspect of the hindfoot. Clinical and cadaveric studies have reported that achievement of at least 10 mm of displacement can change ankle contact pressures and joint distribution [5, 8].

To achieve an adequate displacement of the posterior calcaneus, an open procedure is traditionally performed through the skin incision in line with the osteotomy [11, 17]. Common complication of this procedure includes delayed wound healing, wound infection, and sural nerve damages [1, 3, 16, 20]. The sural nerve is vulnerable to injury during the initial incision because it runs superficially along the lateral aspect of the calcaneus. Evidence of iatrogenic sural nerve-related symptoms following open approach for MDCO have been reported at 6.8–25% [1, 3, 4, 16, 20]. The minimally invasive calcaneal osteotomy (MICO) technique has recently emerged to reduce the incidence of wound complications; nevertheless, the sural nerve may be exposed to greater risk of iatrogenic injury because of limited visualization.

A cadaveric study by Talusan et al. [19] defined extension lines from the calcaneal tuberosity to the plantar fascia origin as landmarks for the identifying safe zone to minimize sural nerve damage in MDCO. Other authors described the position of the skin incision using the sural nerve relationship to the Achilles tendon, with the lateral malleolus as a reference point [2, 18, 21]. However, it is somewhat insufficient to determine the optimal incision area to minimize damage to the sural nerve with only one landmark or two reference points in both open and minimally invasive medial displacement osteotomies. Only one recent cadaveric study has described the course of the sural nerve as the relationship of four anatomical reference points [7].

Therefore, we aimed to describe the anatomical course of the sural nerve in relation to the easily identifiable landmarks at the time surgery and verify the relationship between sural nerve and reference points. We hypothesized that a practical reference area could be provided to minimize injury of the sural nerve during MDCO, regardless of the incision methods.

## Materials and methods

The cadavers used in the present study were donated to our institutions with consent for education and research. In addition, this study was approved by the Ethics Committee of Chuncheon Sacred Heart Hospital, Hallym University (Institutional Review Board number: 2017-95).

Twenty foot and ankle specimens in adult formalin-fixed cadavers were dissected. Of the 20 specimens, 10 (50%) were from females and 10 (50%) were from males. The mean age of the donors at death was 73.9 (range, 49–91) years. The lateral aspect of the foot of all cadavers had intact skin with no signs of previous trauma or surgery, obvious deformities, and/or ulcers.

**Fig. 1:**
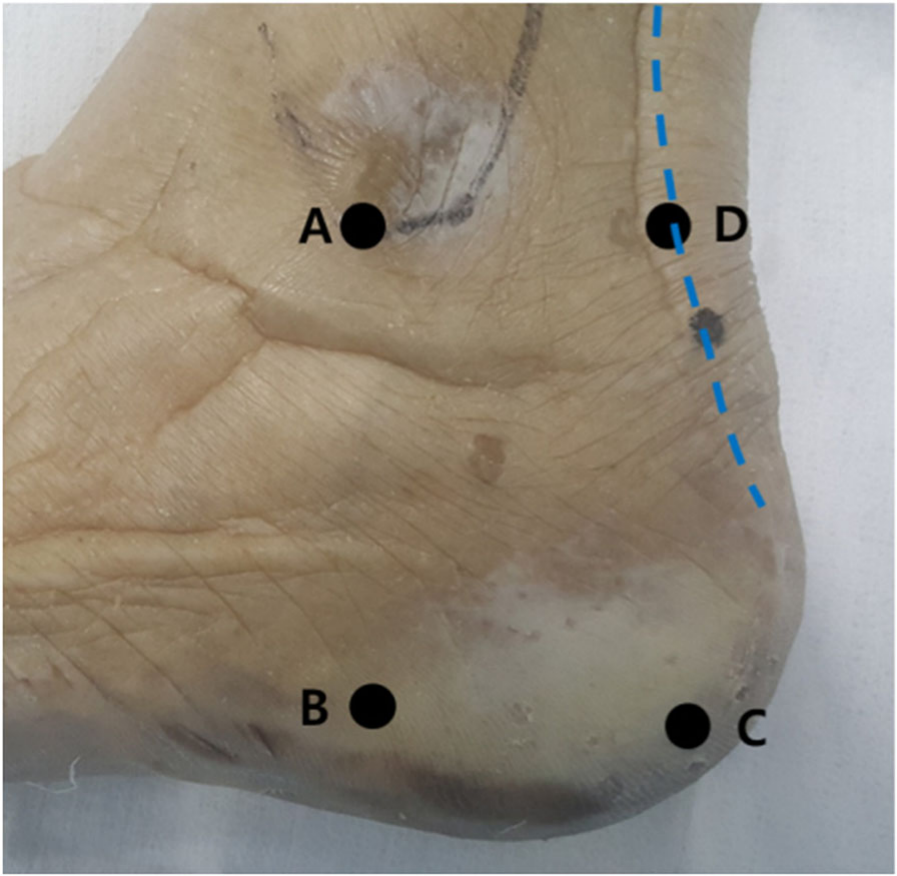
Four reference points on the skin marked before dissection. Point A, the tip of the lateral malleolus; point B, the inferior margin of the calcaneus on the vertical line through point A; point C, the posteroinferior apex of the calcaneus; and point D, the lateral border of the Achilles tendon (blue dotted line) on the horizontal line through point A

The foot and ankle specimens were stabilized in the lateral position, and the following four reference points described by Geng et al. [7] were identified by palpation and marked before dissection: point A, the tip of the lateral malleolus; point B, the inferior margin of the calcaneus on the vertical line through point A; point C, the posteroinferior apex of the calcaneus; and point D, the lateral border of the Achilles tendon on the horizontal line through point A (Fig. 1).

A section of the skin was meticulously dissected from the lateral side of the ankle and hindfoot. The posterior inferior margin of the skin dissection was started at the most inferior and posterior points of the calcaneus. Then, the skin and subcutaneous tissue of the lateral part of the ankle and hindfoot were carefully dissected. This approach provided adequate visibility to confirm the anatomical structure around the sural nerve, including the main trunk of the sural nerve, the lateral calcaneal branches, and the small saphenous vein running along with the sural nerve.

**Fig. 2:**
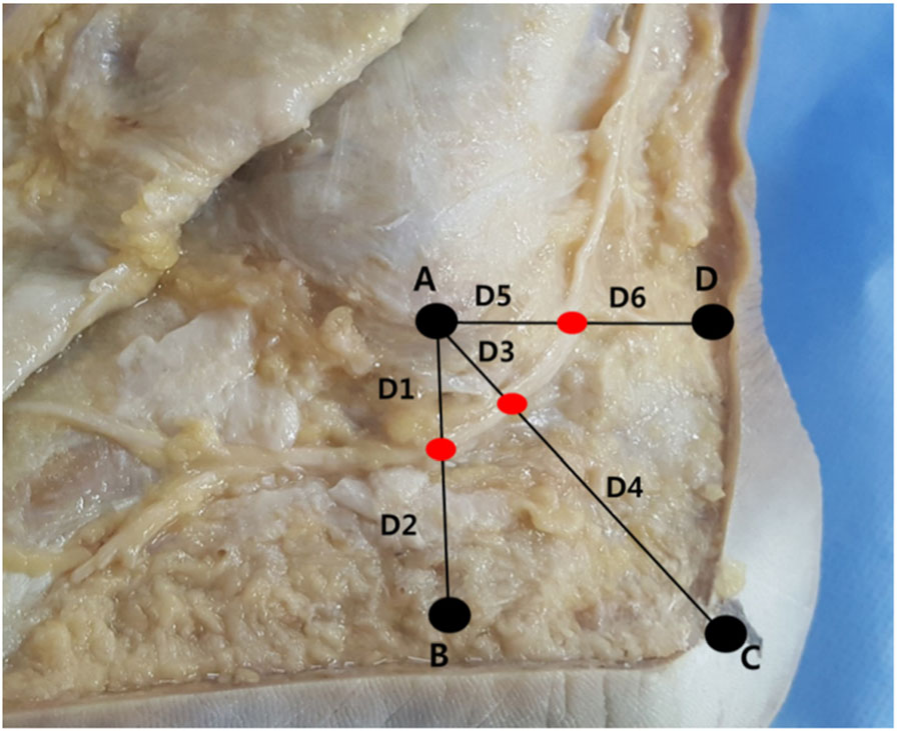
The distances were measured. Line D1, the vertical distance from point A to the sural nerve; line D2, the vertical distance from the sural nerve to point B; line D3, the diagonal distance from point A to the sural nerve; line D4, the diagonal distance from the sural nerve to point C; line D5, the horizontal distance from point A to the sural nerve; and line D6, the horizontal distance from the sural nerve to point D

After a careful dissection, the distances from the sural nerve to points A and B in the vertical direction (lines D1 and D2, respectively), to points A and C in the diagonal direction (lines D3 and D4, respectively), and to points A and D in the horizontal direction (lines D5 and D6, respectively) were measured using a surgical ruler (Fig. 2). The identification of landmarks and measurements of the distances were performed independently by two researchers. Each independent researcher repeatedly measured the distance twice after identifying the landmark. The averages of the two researchers’ measurements were adopted as the values to describe each specimen.

### Statistical analysis

Inter- and intraobserver reliabilities for all measurements were calculated using the intraclass correlation coefficient (ICC). To evaluate the central tendency of all specimens, each distance was presented using the median and range. The ratios were calculated, and each ratio was presented as the median, range, and interquartile range. A Spearman correlation analysis was performed to determine the relationship between the measurements. We used scatter plots to visually represent the relationships. The Wilcoxon-Mann-Whitney test was performed to compare the ratio between males and females, while the Spearman correlation analysis was performed to evaluate the relationship between ratio and age. All statistical analyses were performed using SPSS 25.0 software (SPSS, Chicago, IL), and *P* values less than .05 were considered statistically significant.

## Results

**Fig. 3:**
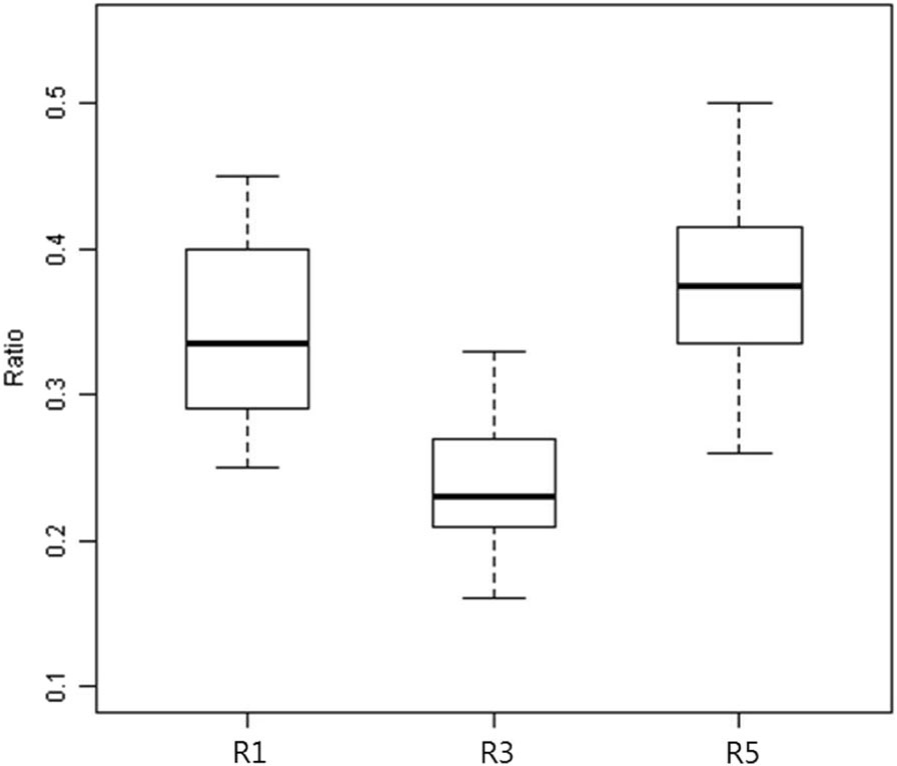
Box plot of the all ratios (*n* = 20 specimens). The boxes represent the interquartile ranges; the thick horizontal line inside the boxes represents the median values; the horizontal lines above and below the boxes represent the ranges. R1, ratio of D1 to D1+D2; R3, ratio of D3 to D3+D4; R5, ratio of D5 to D5+D6

Intraclass correlation coefficients were generated for all measurements. All measurements were greater than 0.8 (indicating acceptable reliability) and were employed in the study. The median values, ranges, and interquartile ranges (IQR) for all distances measured between the sural nerve and the selected reference landmarks are listed in Table 1. Mean values and standard deviations were also provided to help compare with the distances reported in previous studies. The median ratios of D1 to D1+D2, D3 to D3+D4, and D5 to D5+D6 were 0.34 (range 0.25 to 0.45), 0.23 (range 0.16 to 0.33), and 0.38 (range 0.26 to 0.50), respectively (Table 2). The median values, ranges, and IQR for all ratios are also shown in a box plot (Fig. 3).

**Table 1:** The distances measured between the sural nerve and the selected reference landmarks (*n* = 20 specimens)

Distance (mm)	Median	Range		IQR		Mean	SD
		Min	Max	Q1	Q3		
D1	13.00	10.00	21.00	11.25	18.00	14.45	3.78
D2	27.50	21.00	32.00	24.00	30.75	27.20	3.71
D3	13.00	9.00	18.00	10.50	14.00	12.90	2.63
D4	41.50	27.00	53.00	35.50	46.50	41.45	6.89
D5	15.00	8.00	24.00	12.25	19.75	15.65	4.53
D6	27.50	13.00	39.00	20.50	30.00	25.90	6.56

D1, the vertical distance from point A to the sural nerve; D2, the vertical distance from the sural nerve to point B; D3, the diagonal distance from point A to the sural nerve; D4, the diagonal distance from the sural nerve to point C; D5, the horizontal distance from point A to the sural nerve; D6, the horizontal distance from the sural nerve to point D; IQR, interquartile ranges; Q1, lower quartile; Q3, upper quartile; SD, standard deviation

**Table 2:** The ratios calculated based on the measured distance values (*n* = 20 specimens)

Ratio	Median	Range		IQR	
		Min	Max	Q1	Q3
R1	0.34	0.25	0.45	0.29	0.40
R3	0.23	0.16	0.33	0.21	0.27
R5	0.38	0.26	0.50	0.33	0.42

R1, ratio of D1 to D1+D2; R3, ratio of D3 to D3+D4; R5, ratio of D5 to D5+D6; IQR, interquartile ranges; Q1, lower quartile; Q3, upper quartile

**Fig. 4:**
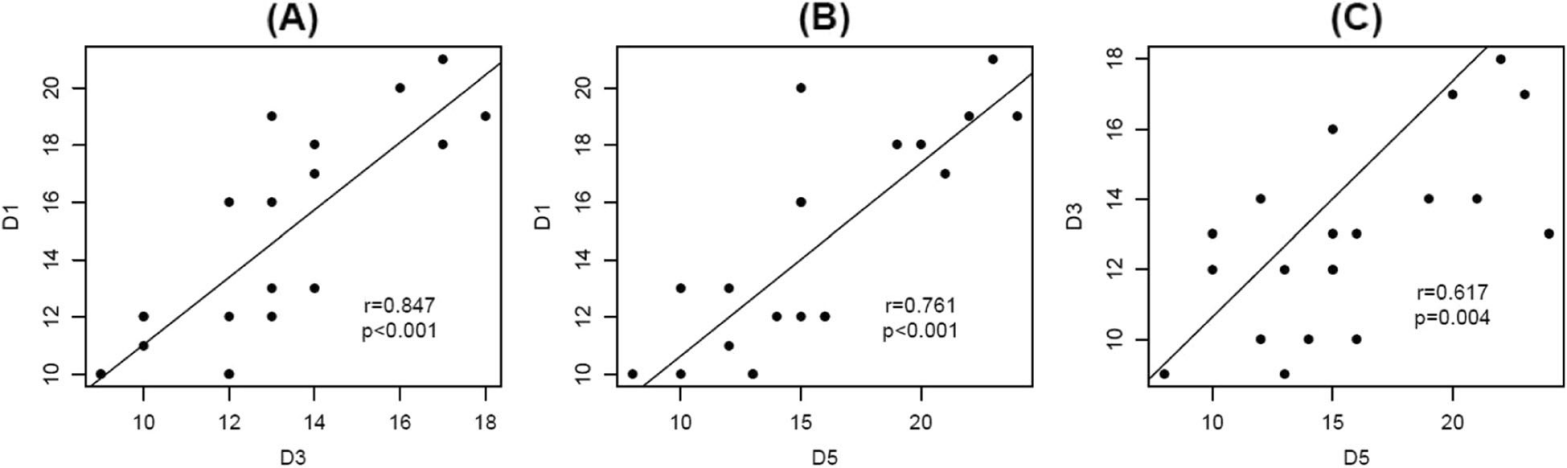
Scatter plot to visually represent the relationships between the distances. D1, the vertical distance from the lateral malleolar tip to the sural nerve; D3, the diagonal distance from the lateral malleolar tip to the sural nerve; D5, the horizontal distance from the lateral malleolar tip to the sural nerve

**Fig. 5:**
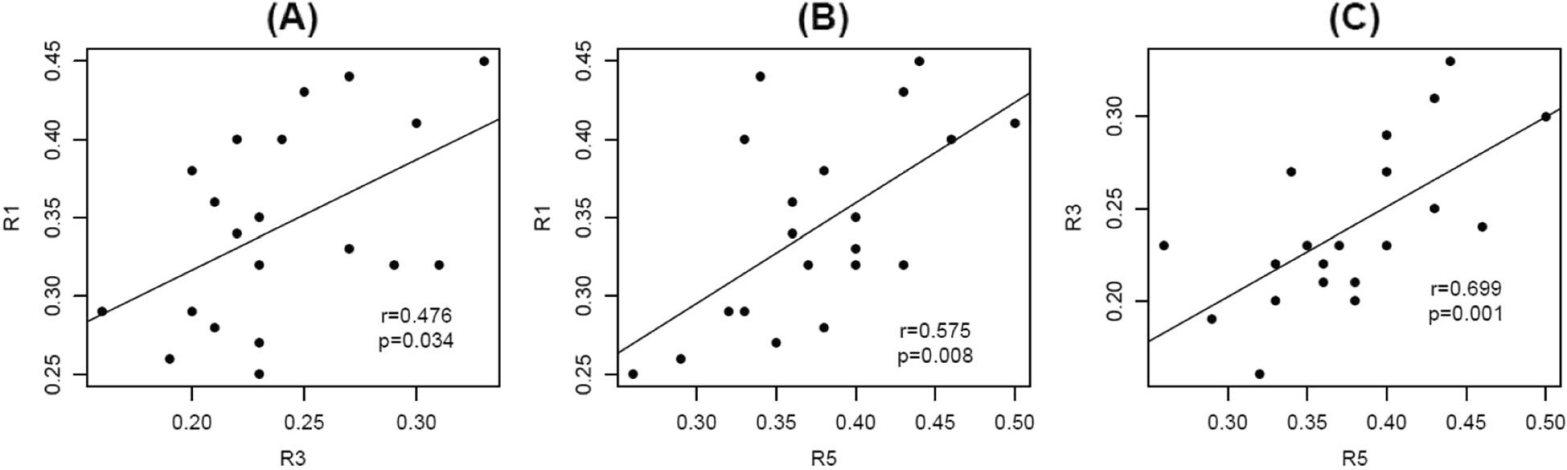
Scatter plot to visually represent the relationships between the ratios. R1, ratio of D1 to D1+D2; R3, ratio of D3 to D3+D4; R5, ratio of D5 to D5+D6

The distance values measured from the lateral malleolar tip to the sural nerve in each direction of the reference points showed a statistically significant correlation with each value and a relatively high correlation coefficient (Fig. 4). All ratios (R1, R3, and R5) showed statistically significant correlations with each ratio and relatively high correlation coefficients (Fig. 5).

There was no significant difference in all ratios by gender (*p* = 0.579 [R1], 0.280 [R3], and 0.165 [R5]). The correlation between age and all ratios was not significant (*p* = 0.462 [R1], 0.221 [R3], and 0.163 [R5]).

## Discussion

Medial displacement calcaneal osteotomy (MDCO) is commonly used concurrently with other osteotomies and tendon transfers in the treatment of adult acquired flatfoot deformity (AAFD). Traditionally, the procedure includes an oblique incision on the lateral heel. A common complication is sural nerve damage around the incision [1, 16, 20]. Although minimally invasive or percutaneous techniques have recently emerged in the hope of minimizing incisional complications, the risk of iatrogenic damage to the sural nerve remains [6, 9, 19].

Because the sural nerve runs superficially on the lateral aspect of the heel [14], it is theoretically vulnerable to injury during the incision if an inappropriate incision is made. In order to minimize damage to the sural nerve in both open and minimally invasive/percutaneous medial displacement osteotomies, it is of utmost importance that an adequate skin incision is made. In our study, the reference points of the anatomical structure that can be easily recognized on the skin surface during surgery were described in relation to the anatomical course of the sural nerve.

Previous studies have also detailed the sural nerve in relation to anatomical landmarks. Solomon et al. [18] used two points corresponding to the tip and the most prominent posterior part of the lateral malleolus as reference points. Kammar et al. [10] presented four arbitrary points as references at a certain distance from the point of insertion of the Achilles tendon into the calcaneus. Talusan et al. [19] defined extension lines from the calcaneal tuberosity to the plantar fascia origin as the baseline. We believe that these reference points (only two points, arbitrary points on the Achilles tendon and a single baseline) are insufficient to provide detailed reference information to determine the optimal incision site to avoid damage to the sural nerve during MDCO. We believe the four landmarks applied in this study would be more useful in describing the course of the sural nerve.

**Fig. 6:**
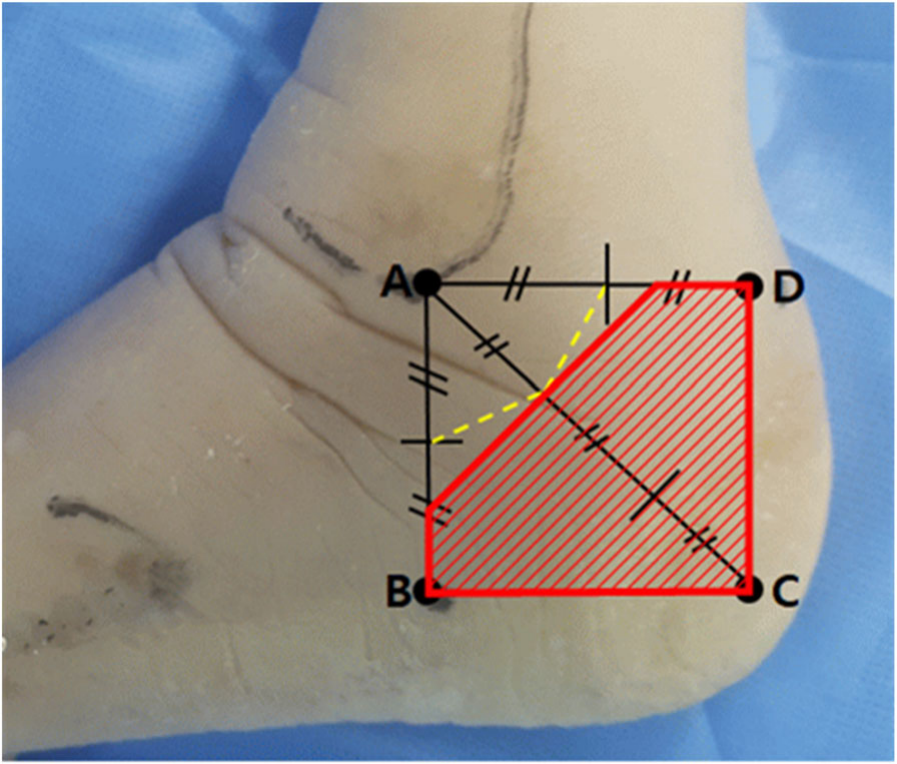
The incision area for minimizing sural nerve injury in MDCO. The yellow dotted line represents the baseline of distance from the lateral malleolar tip to the sural nerve. The red hatched area represents a practical zone for clinical applications, including more than one half of the horizontal distance between points A and B, more than one half of the vertical distance between point A and D, and more than one third of the diagonal distance between point A and C

We used a total of four reference points (the tip of the lateral malleolus, the inferior margin of the calcaneus, the posteroinferior apex of the calcaneus, and the lateral border of the Achilles tendon) that can be easily recognized on the skin surface during surgery. In previous studies [2, 7, 18], the mean vertical distances between the tip of the lateral malleolus and the sural nerve were reported as 13.15 ± 6.88, 13 ± 7, and 13.1 ± 4.1 mm, respectively, compared with 13 ± 3.78 mm in our study. These differences in values were not significant and may be accounted for by the size and race of the cadavers and the variability of the nerve course. We therefore determined that it would be more reliable and clinically useful to describe the ratio rather than the absolute distance. Table 2 and Fig. 3 show that the maxima of the ratios (R1, R3, and R5) approximate to 0.45, 0.33, and 0.50, respectively. Using these maximum ratio values through reference landmarks based on the tip of the lateral malleolus prior to surgical incision, we believe that an incision area for sural nerve preservation can be established during MDCO (Fig. 6).

Geng et al. [7] reported the maximum of the ratios applied in the same manner as this study, with slightly different values except for R3. The authors suggested that skin incision for MDCO can be determined using a reference point that is one third (or not exactly 32%) of the distance from the tip of the lateral malleolus to the posteroinferior margin of the calcaneus; however, the use of vertical and horizontal lines between reference points was not mentioned. Our results showed that both distance and ratio values measured from the lateral malleolar tip to the sural nerve in each direction of the reference points had significant correlations with each value. Therefore, we believe that this study, presenting up to auxiliary reference points in the vertical and horizontal directions, would more accurately account for the incision criteria that minimize the damage of the sural nerve during MDCO.

In open procedures for MDCO, the oblique incision may be made equal to the osteotomy line at the back of more than one third of the line connecting the posteroinferior apex of the calcaneus from the tip of the lateral malleolus within the proposed incision criteria. Lee et al. [12] discussed the importance of selecting the entry point for the burr in minimally invasive procedures for MDCO; however, they did not propose an accurate entry point. Therefore, the proposed incision criteria in this study can be useful for minimizing iatrogenic damage to the sural nerve in both open and minimally invasive/percutaneous medial displacement osteotomies. Furthermore, there was no significant difference in the results of this study by gender. Madhavi et al. [13] reported that there was no association between sural nerve distribution type and gender in the foot. Therefore, it would be possible to apply the proposed incision criteria in this study regardless of gender.

This study has some limitations. Differences in the incision area for minimizing sural nerve injury between embalmed cadaver and fresh cadaver or live human may exist due to stiffness of soft tissues and immobile joints in embalmed cadaver specimens. However, the cadaveric nature of this study did not allow us to detect such potential differences. Because the ankles of all embalmed cadaveric specimens were fixed at a neutral position of 90°, the measurement between the bony landmarks and the sural nerve was almost identical. The small sample size may have weakened the statistical power with respect to the possibility of sural nerve variations. For this reason, only the main trunk of the sural nerve was analyzed, and there may be an anatomical variation of the branches of the sural nerve. Larger samples are necessary for future studies to generate more accurate descriptions of the sural nerve. Furthermore, to clinically apply the results of this study, a validation study using a larger number of cadavers is required.

## Conclusions

The distance ratios presented in this cadaveric study can be helpful to describe the course of the sural nerve according to easily identifiable bony landmarks during MDCO. We suggested skin incision criteria of more than one third of the distance from the tip of the lateral malleolus to the posteroinferior apex of the calcaneus as the main reference, and more than 1/2 points from the tip of the lateral malleolus to the vertical and horizontal directions would be the auxiliary reference to minimize iatrogenic damage of the sural nerve during MDCO. Furthermore, the proposed incision areas are more practical reference points for minimizing sural nerve injury in both open and minimally invasive/percutaneous MDCO.

## Supplementary information

**Additional file 1:** The datasets used and analyzed during the current study.

## Data Availability

The datasets used and analyzed during the current study are presented in Additional file 1.

## Abbreviations

AAFD: Adult acquired flatfoot deformity
ICC: Intraclass correlation coefficient
IQR: Interquartile ranges
MDCO: Medial displacement calcaneal osteotomy
MICO: Minimally invasive calcaneal osteotomy
